# Supplementary material for: Deep mutational scanning of the human insulin receptor ectodomain to inform precision therapy for insulin resistance
Source: Nat Commun. 2025 Oct 15;16:9143. doi: 10.1038/s41467-025-64178-4 (PMC12528385; doi:10.1038/s41467-025-64178-4)
Supplement: Supplementary file 1 — Supplementary Information [file 41467_2025_64178_MOESM1_ESM.pdf]

**number of ccs reads (HiFi Reads)**	4,016,907
**mean ccs readlength**	3,041
**median ccs read quality**	Q49
**mean number of passess (subreads per ccs)**	20
**ccs aligned to the amplicon**	3,568,566
**low quality ccs (filtered)**	309,112
**ccs unmapped to the amplicon**	139,229
**average (median) ccs per barcode**	19.15
**total number of barcodes with consensus sequences**	134,800
**barcodes with consensus sequences (no_indels with zero or single aa changes)**	126,227
**barcodes with single_aa_substitutions (nonsynonymous)**	80,972
**barcodes with single_aa_substitutions (stop codon)**	3,539
**barcodes with synonymous mutations**	4,023
**barcodes with WT sequence, no mutagenesis**	41,232
**unique missense aa substitutions in the library (library completeness)**	15,996 (out of 18,560 (20*928) possible)
**unique stop variants in the library**	813
**missense aa substitutions associated with single barcode**	3,032
**average number of variants detected after FACS sorting**	13,638
**number of missense single aa substitutions linked with only one barcode**	3,031
**number of missense single aa substitutions linked with two or more barcodes**	12,965

Supplementary Table 1: Summary Statistics from PacBio sequencing of Barcoded Plasmid Variant Library. ccs reads = single molecule consensus reads; HiFi reads = ccs reads with predicted accuracy >Q20; aa = amino acid; FACS = Fluorescence-activated

Site 1/1' residues		
Gly37	Arg298	Glu602
Met38	Gln299	Arg603
Asp39	Gly300	Arg604
Arg41	Cys301	Leu723
Asn42	His302	Glu724
Asn43	Glu343	Glu725
Arg46	Gly373	Ser726
His59	Gly374	Ser727
Gln61	Asn375	Phe728
Leu63	Asn376	Arg729
Leu 64	Arg399	Lys730
Phe66	Ser400	Phe732
Lys67	Tyr401	Glu733
Tyr87	Trp520	Asp734
Leu89	Pro521	Tyr735
Phe91	Pro522	Leu736
Arg92	Asp523	His737
Tyr94	Phe524	Asn738
Phe115	Arg525	Val739
Phe116	Asp526	Val740
Tyr118	Leu527	Phe741
Val121	Leu 528	Val742
Phe123	Arg566	Pro743
Glu124	Ser567	Arg744
Phe145	Asn568	Pro745
Glu147	Asp569	Ser746
Lys148	Pro570	Arg747
Arg215	Lys571	Lys748
Lys294	Val597	Arg749
Asn295	Thr598	Arg750

Site 2/2' residues		
Asp178	Phe530	Leu583
Asn179	Val559	Asn698
Glu180	Ile561	Gln699
Glu181	Asp562	Ser700
Tyr504	Pro563	Glu701
Arg506	Pro564	Tyr702
Thr507	Leu565	Glu703
Ser508	Gln573	Asp704
Asp510	Asn574	Ser705
Lys511	His575	Ala706
Ile512	Pro576	Gly707
Leu513	Gly577	Glu708
Leu514	Trp578	Cys709
Arg515	Leu579	Pro713
Trp516	Arg581	Leu714
Gly529	Gly582	

Supplementary Table 2: Numbers of residues potentially contributing to Insulin-binding sites 1/1' and 2/2', curated from structural studies

Variant	Median expression score at corresponding position (N) [LoF cut off -0.26]*	Median binding score at corresponding position (N) [LoF cut off -0.15]*	Median signalling score at corresponding position (N) [LoF cut off -0.10]*
C293R	-0.38 (13)	-0.19 (13)	-0.04 (13)
T211I	0.01 (14)	-0.35 (14)	-0.15 (14)
R279H	-0.13 (14)	-0.23 (14)	-0.03 (14)
N458D	-0.53 (12)	-0.17 (12)	-0.22 (12)
V93A	-0.30 (9)	-0.64 (9)	-0.14 (9)
N489D	-0.15 (17)	-0.28 (17)	-0.07 (17)
D523N	-0.27 (18)	-0.48 (18)	-0.31 (18)
Y606C	0.036 (10)	-0.26 (10)	-0.14 (10)
W642R	-1.09 (7)	-0.67 (6)	-0.36 (9)
S835I	-0.47 (13)	-0.38 (13)	-0.69 (15)
V867F	-0.36 (15)	-0.34 (15)	-0.10 (15)

Supplementary Table 3: Median function scores of all missense variants at positions where a pathogenic mutation has been reported, but where no data were generated for that specific variant. The number of missense variants with scores at each position is recorded after scores in brackets

Target	Species	Supplier	Catalogue No.	Use	Concentration/ Comment
Human INSR (mAb 83-7)	Mouse	Prof Kenneth Siddle (81)	N/A	FACS	100 nmol/L
Human INSR (mAb 83-14)	Mouse	Prof Kenneth Siddle (81)	N/A	FACS	100 nmol/L
AlexaFluor 647-conjugated anti-Phospho-Akt (Ser473/474)	Rabbit	CST	4075	FACS	1:200
Phospho-IGF-I Receptor β (Tyr1135/1136)/Insulin Receptor β (Tyr1150/1151) (19H7)	Rabbit	CST	3024	WB	1:1000
Insulin Receptor β (4B8)	Rabbit	CST	3025	WB	1:1000
Phospho-Akt (Ser473) (D9E) XP®	Rabbit	CST	4060	WB	1:1000
Akt (pan) (40D4)	Mouse	CST	2920	WB	1:1000
Phospho-p44/42 MAPK (Erk1) (Tyr204)/(Erk2) (Tyr187) (D1H6G)	Mouse	CST	5726	WB	1:1000
p44/42 MAPK (Erk1/2) Antibody	Rabbit	CST	9102	WB	1:1000
Anti-mouse IgG, HRP-linked Antibody	Horse	CST	7076	WB	1:10,000
Anti-rabbit IgG, HRP-linked Antibody	Goat	CST	7074	WB	1:5000
Myc-Tag (9B11)	Mouse	CST	2276	WB	1:1000
β-Actin Antibody	Rabbit	CST	4967	WB	1:1000
IGF-I Receptor β (D23H3) XP®	Rabbit	CST	9750	WB	1:1000

Supplementary Table 4: Commercial Antibodies used in this study. CST = Cell Signaling Technologies; WB = Western blotting

MaveDB URN	Assay	Direct Link
urn:mavedb:00001239-a-7	Cell Surface Expression	https://www.mavedb.org/score-sets/urn:mavedb:00001239-a-7
urn:mavedb:00001239-a-6	Insulin Binding	https://www.mavedb.org/score-sets/urn:mavedb:00001239-a-6
urn:mavedb:00001239-a-1	Insulin Signalling	https://www.mavedb.org/score-sets/urn:mavedb:00001239-a-1
urn:mavedb:00001239-a-2	mAb 83-07 Binding	https://www.mavedb.org/score-sets/urn:mavedb:00001239-a-2
urn:mavedb:00001239-a-3	mAb 83-14 Binding	https://www.mavedb.org/score-sets/urn:mavedb:00001239-a-3
urn:mavedb:00001239-a-4	mAb 83-14 Signalling	https://www.mavedb.org/score-sets/urn:mavedb:00001239-a-4
urn:mavedb:00001239-a-5	mAb 83-07 Signalling	https://www.mavedb.org/score-sets/urn:mavedb:00001239-a-5

Supplementary Table 5: MaveDB URN and Direct Links to interrogatable datasets from each assay, available in the MaveDB Respository

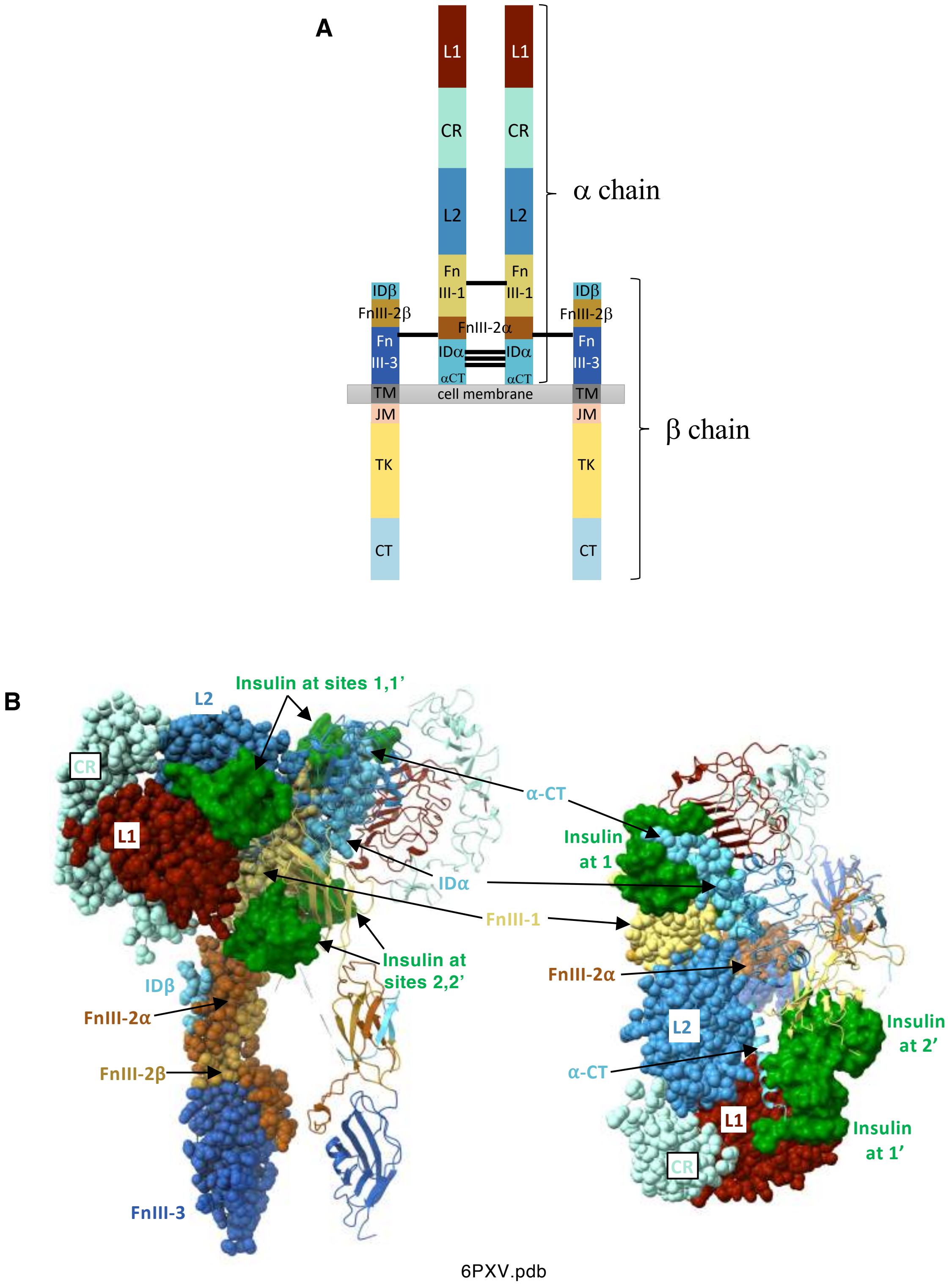

Supplementary Figure 1: Schematic of INSR with coloured domains and cryoEM structure coloured by domain. A, Schematic of INSR with coloured domains **B**, cryoEM structure of the ectodomain bound to 4 insulin molecules (PDB 6PXV) coloured by domain using the same colour scheme. The *en face* view is shown on the left, and the view from above on the right. Image generated using UCSF ChimeraX (78)

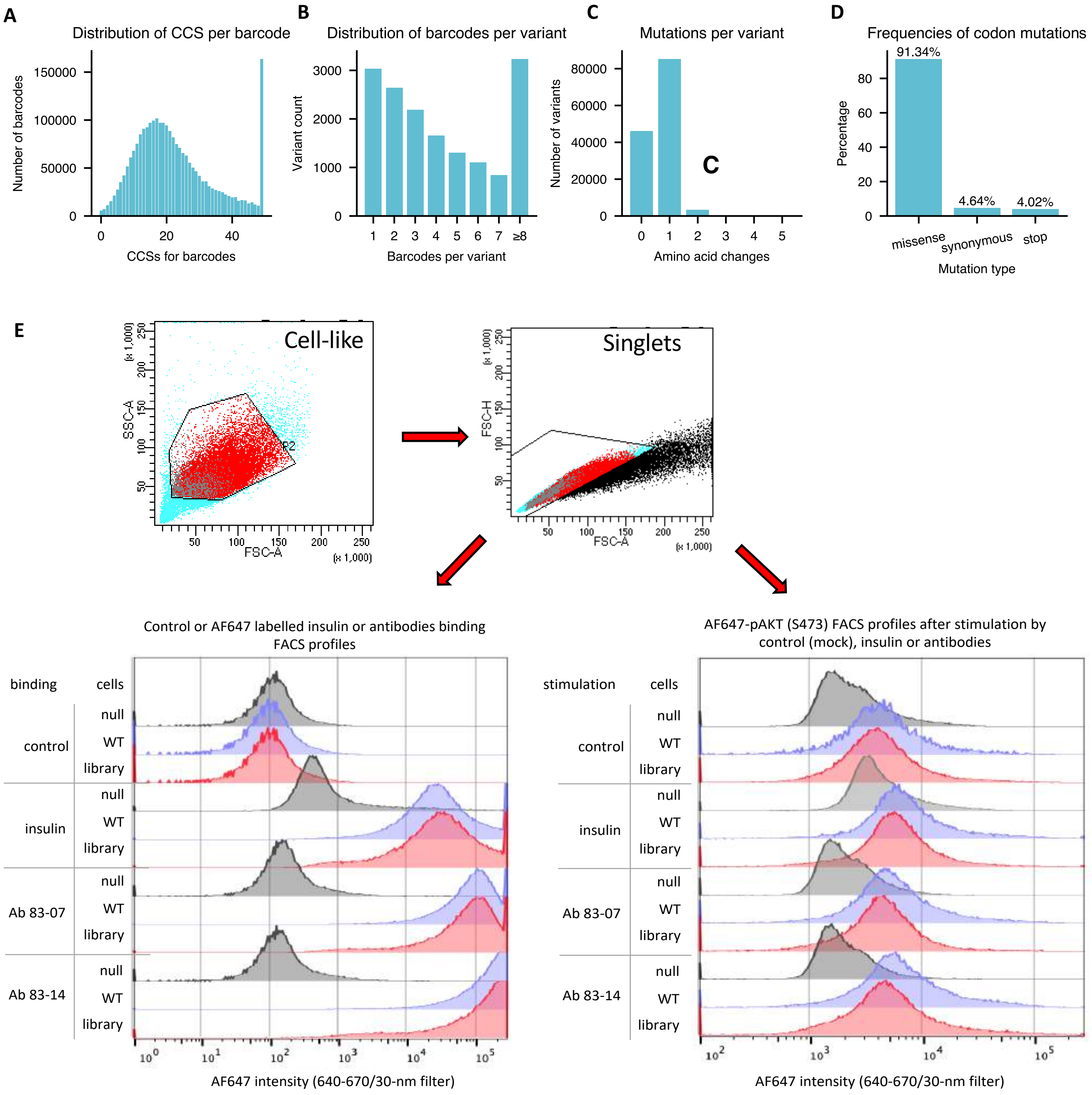

Supplementary Figure 2: Characteristics and Validation of Barcoded Plasmid and Cellular Variant Libraries
A, Distribution of PacBio circular consensus reads (CCS) per barcode. **B**, Distribution of barcodes per variant. **C**, Mutations per variant showing that most INSR cDNAs in the plasmid library have either one amino acid change (a missense mutation), or no amino acid change. **D**, Frequencies of missense, synonymous and stop mutations in the plasmid library. **E**, FACS gating strategy and FACS profiles for insulin or antibody binding (left panel) or stimulated signalling (right panel) for null (grey), wild type (blue) and library cells (red).

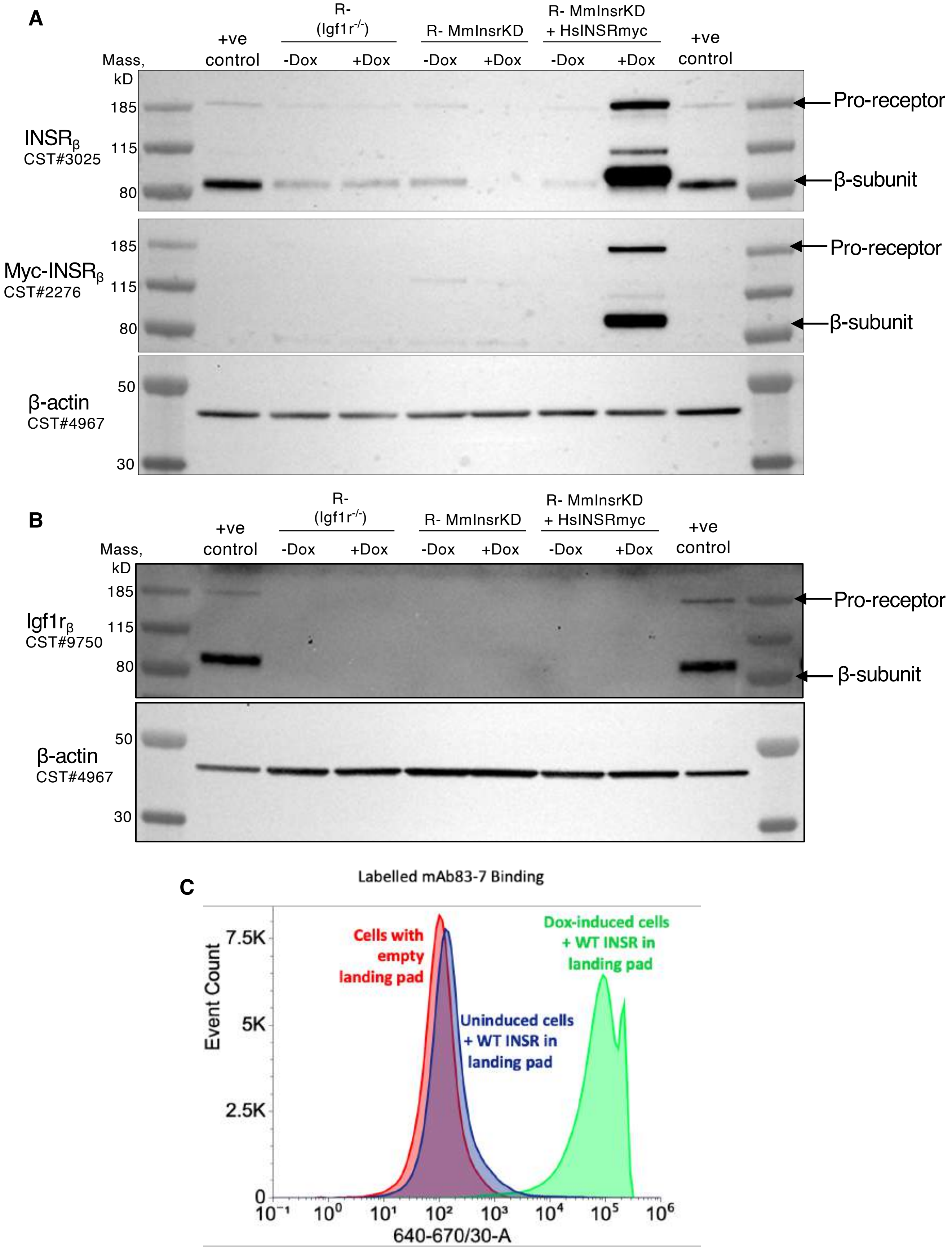

Supplementary Figure 3: Validation of Cellular Model used for Massively Parallel Functional Assays:
A. Immunoblots demonstrating downregulation of endogenous mouse Insr and comcomitant induction of myc-tagged human INSR on exposure of R-MmInsrKD + HsINSRmyc mouse embryo fibroblasts (MEFs harbouring inducible WT human myc-INSR and anti-mouse Insr shRNA) to doxycycline. Controls are R- (Igf1r-/-) MEFs, R- MmInsrKD MEFs harbouring inducible anti-Insr shRNA only, and Hepa1-6 cells as positive controls. Dox = doxycycline. **B**. Immunoblots demonstrating absent expression of Igf1r in keeping with Igf1r gene knockout. b-actin is shown as a loading control. In this case Hek293T cells were positive controls. **B.** FACS trace demonstrating mAb83-7 binding as a surrogate for human INSR cell surface expression, demonstrating tight control of transgenic myc-INSR expression by doxycycline, with a high dynamic range of the expression assay.

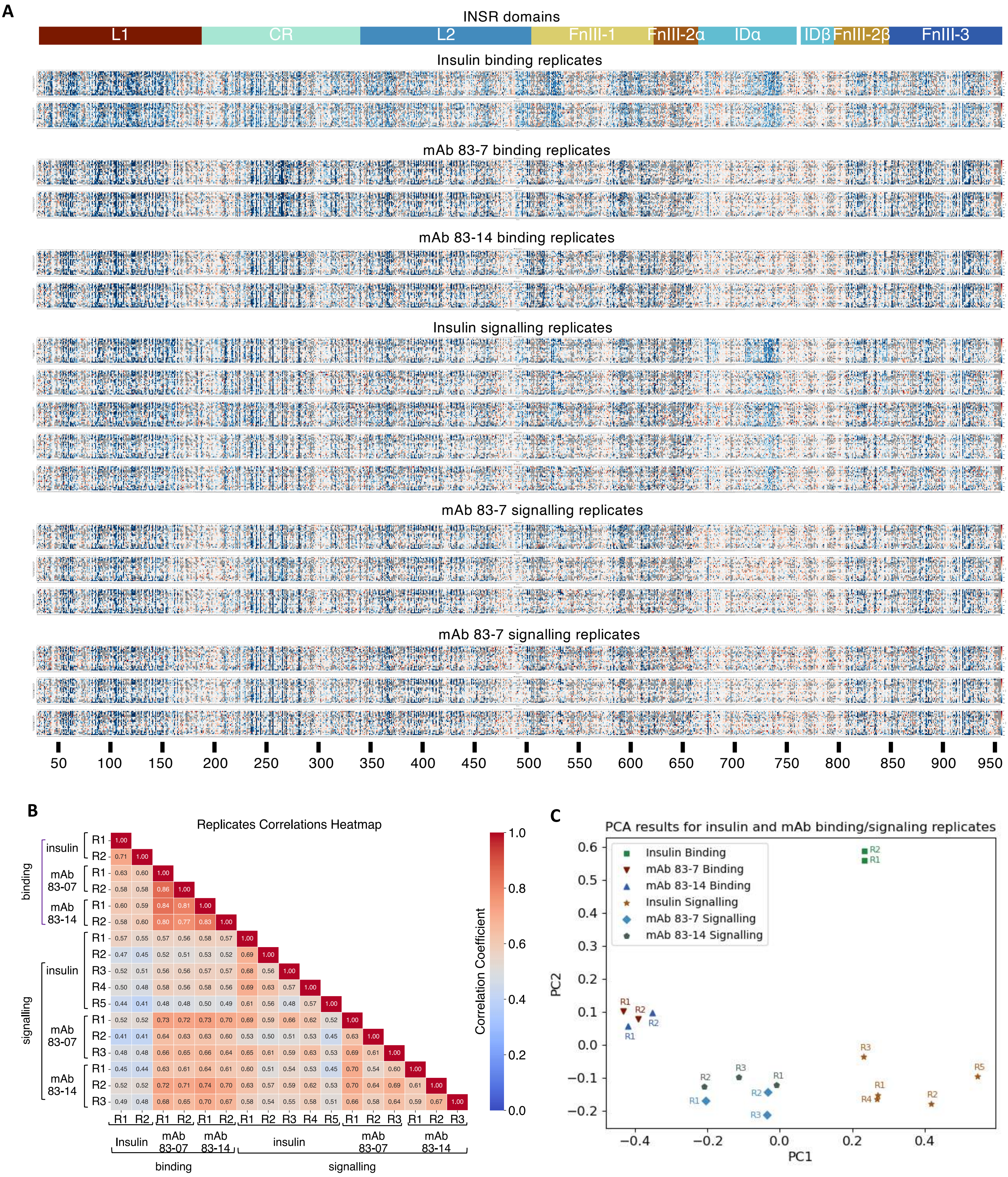

Supplementary Figure 4: Overview of massively parallel assay results and replication A, INSR extracellular domain architecture with variant-function heatmaps for each replicate of insulin, mAb 83-7 and mAb 83-14 binding and signalling assays. **B**, Pearson's correlation matrix showing coefficients of variation for all pairwise replicate combinations. Correlation coefficients (ρ) are annotated inside heatmap cells. **C**, Principal Component Analysis (PCA) of replicates.

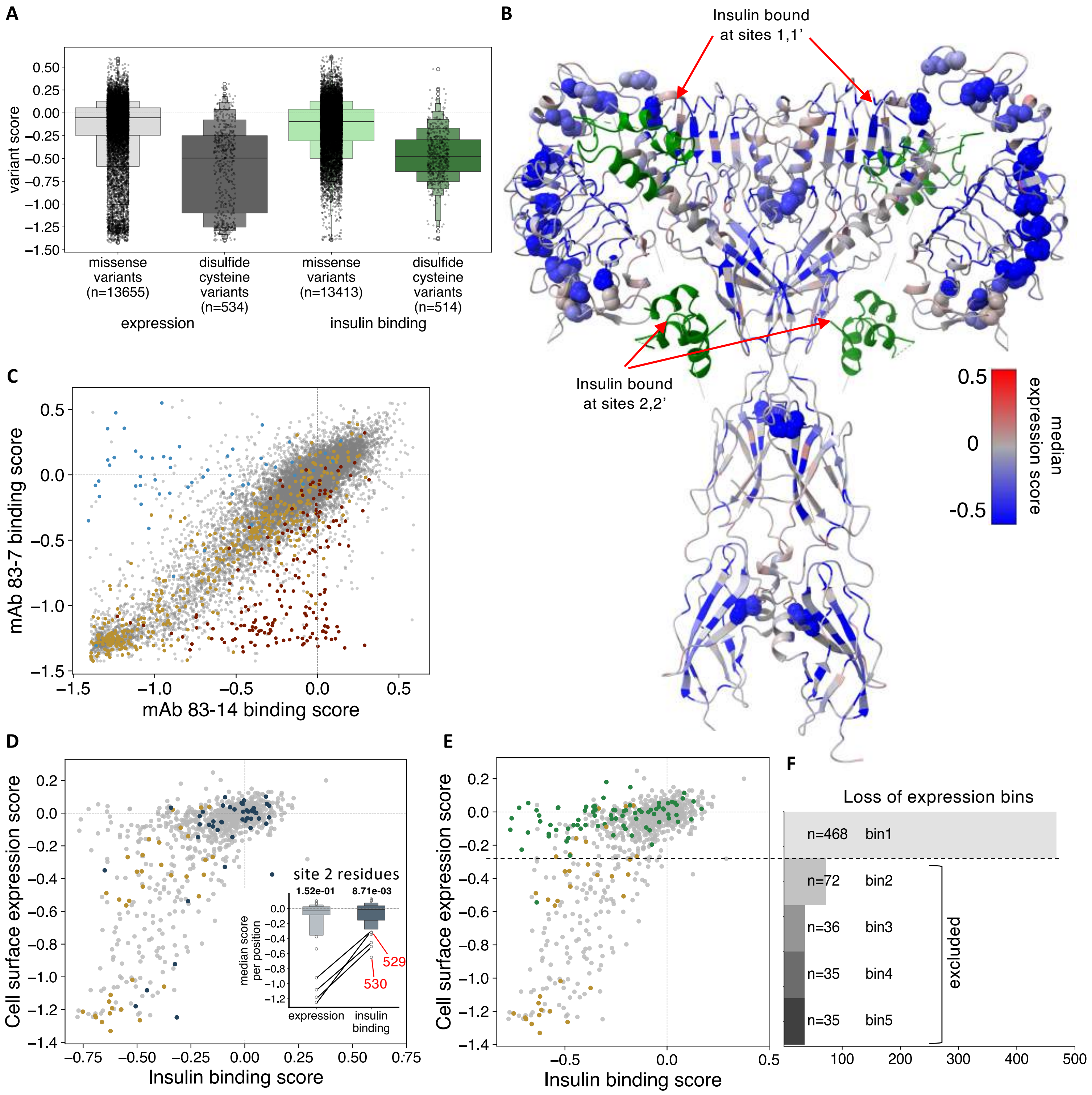

Supplementary Figure 5: Further Description of Massively Parallel Cell Surface Expression and Insulin Binding Assays **A**, Boxenplot comparing cell surface expression and insulin binding variant score distributions for non-cysteine residues with cysteine residues crucial for disulphide bond formation. Inner boxes show interquartile ranges, with median line at centre, with sequential nested boxes showing 50% of remaining datapoints. **B**, Structure of 4 insulin-bound INSR ectodomain (PDB: 6PXV) with residues coloured by median cell surface expression score per position. All cysteine residues are shown in space filling style. **C**, Scatter plot of individual variant scores for mAb 83-7 binding, with dark-brown coloured outliers, and mAb 83-14 binding, with light-blue coloured outliers. Cysteine residues involved in disulphide bond formation are coloured golden brown for reference. **D**, Boxenplot, as in A, of median scores for insulin binding and cell surface expression. Insulin binding site 2 residues are shown in blue and cysteine residues are coloured golden brown for reference. The inset boxplot shows expression and insulin binding scores for insulin binding site 2 residues (n=47). The Mann-Whitney U Test was used to assess whether the insulin binding and expression scores of site 2 residues differ significantly from the distribution of scores of all other residues. Any variants outlying in both assays are connected by lines. Residues 529 and 530, outliers only in the insulin binding assay, are labelled **E**, Scatter plot showing median insulin binding versus cell surface expression scores, with site 1 residues highlighted in green. The horizontal box plot stratifies residues into 5 bins based on expression loss. Residues in the second bin or lower are classified as loss-of-expression variants and were coloured white when colouring the structure in Figure 2G by insulin binding score.

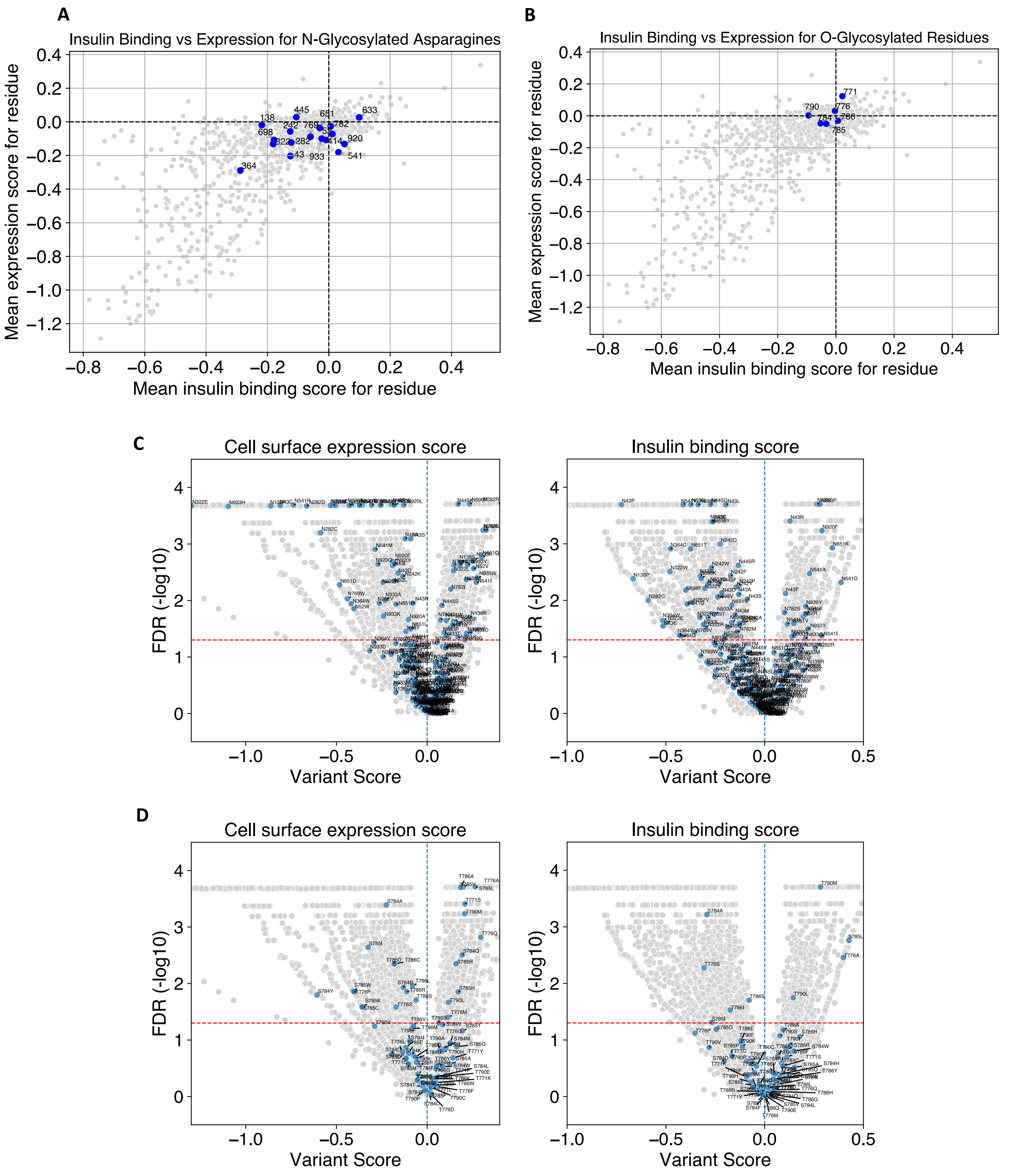

Supplementary Figure 6: Functional Consequences of Mutation of Extracellular Glycosylated Residues
Mean insulin binding vs mean expression scores for all mutations at **A.** N-glycosylated asparagines 43, 52, 138, 242, 282, 322, 364, 414, 445, 541, 633, 651, 698, 769, 782, 920, 933 (identified as N-glycosylation sites in (79)) or **B.** O-glycosylated amino acids (T771, 776, 786, 790; S784,785; confirmed as O-glycosylated residues in (80) **C.** Volcano plot showing effects on expression (left) and insulin binding (right) of individual mutations of N-glycosylated asparagines **D.** Volcano plot showing effects on expression (left) and insulin binding (right) of individual mutations of O-glycosylated amino acids. Variant labels in **C** and **D** are visible on zooming in.

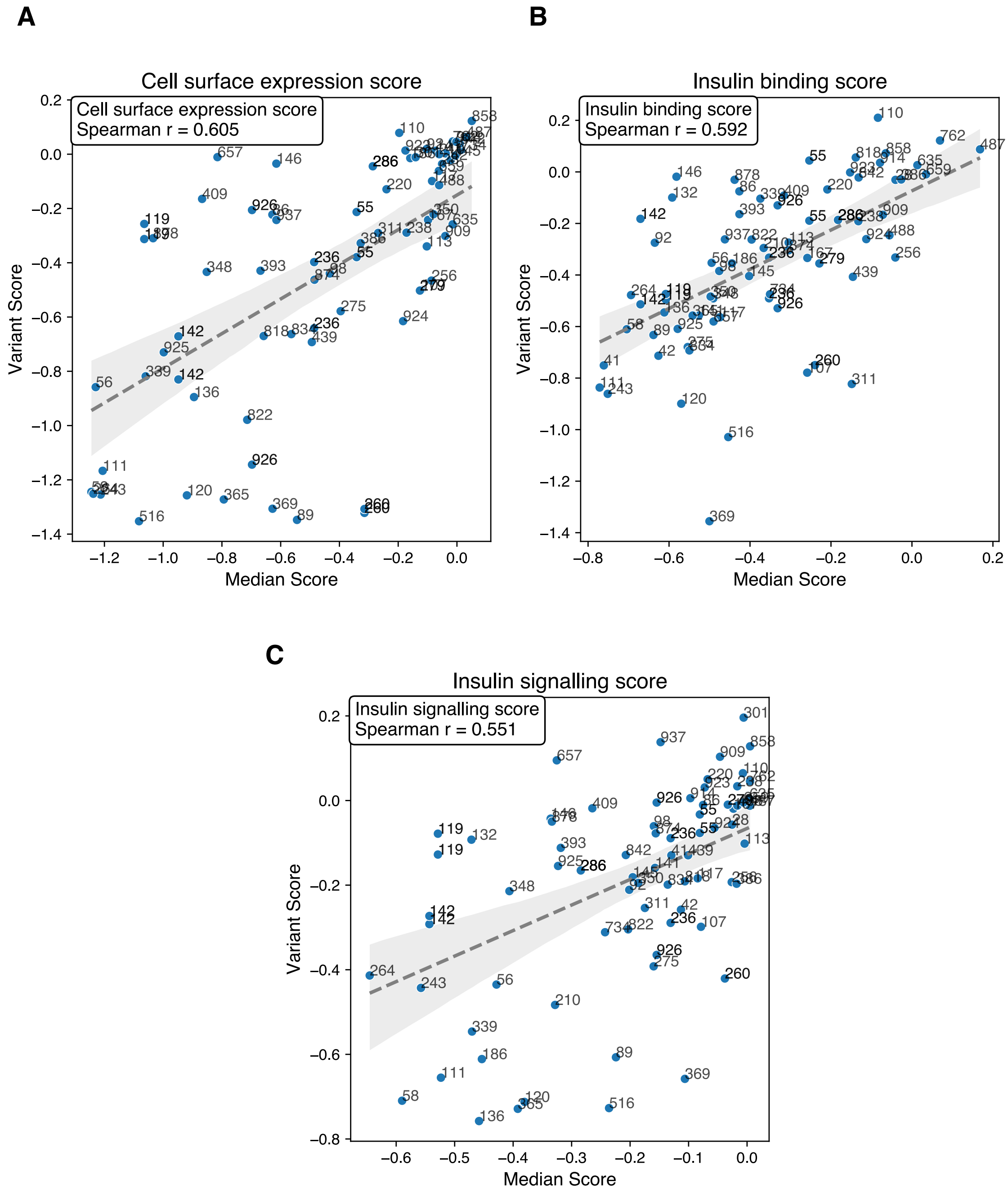

Supplementary Figure 7: Functional Data for all Variants at Sites where Pathogenic Mutations have been Reported but for which no Pathogenic Variant-specific Data were Generated For all 77 reported pathogenic missense mutations in the INSR extracellular domain for which functional data were generated, variant specific scores are plotted against median scores for all variants at the same position for A, cell surface expression B, insulin binding and C, insulin signalling. Spearman's rank correlation tests were undertaken and correlation co-efficients are indicated for each plot.

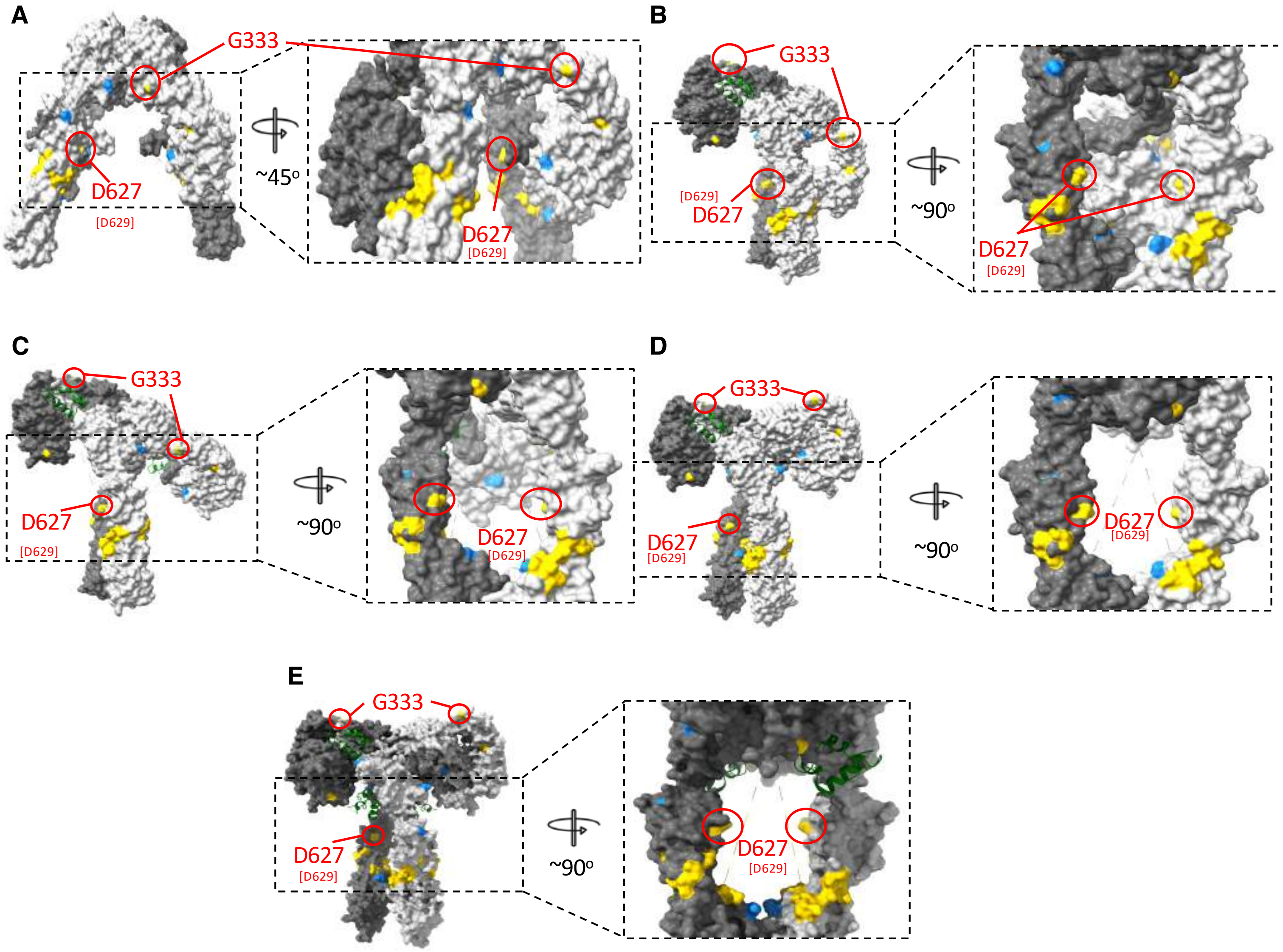

Supplementary Figure 8: INSR residues identified to result in gain of insulin binding upon substitution shown mapped to the cryo-EM structures of various insulin-bound states. Monomers are coloured different shades of grey. Insulin is represented by green ribbon structures. Yellow depicts residues where multiple substitutions increase insulin binding (G183, H274, G333, D627, V631, N633, S634, S635, S636, W659, E660, R661, Q662, A663, E664, D665, S666, E667, S682, R683, T684, S686, P687, P688). Blue depicts residues where a unique substitution increases binding (D169, N541, N568, T653, K679). Some residues highlighted may be involved in interactions with parts of the receptor not resolved in available structures. **A**, Unbound, INSR B-isoform (PDB: 8U4B). The highlighted section is rotated counterclockwise 45o to the full view of the receptor. **B**, Single insulin bound (green ribbon) INSR isoform A (PDB: 7STI). PDB structure 7STI is the A isoform of the *Mus musculus* orthologue, but for consistency residues highlighted are numbered according to the human B isoform pro-receptor with the corresponding mouse residue in brackets. The highlighted section is rotated counterclockwise 90°. **C** and **D,** Two insulin bound (green ribbons) INSR (PDB: 7STJ (asymmetrical), and 7STH (symmetrical)). These structures are the A isoform of the mouse orthologue, but for consistency residues highlighted are numbered according to the human B isoform pro-receptor with the corresponding mouse residue in brackets. Highlighted sections are rotated counterclockwise 90º. **E)** Saturated, four insulin bound INSR (PBD: 6PXV). The highlighted section is rotated counterclockwise 90º to the full view.

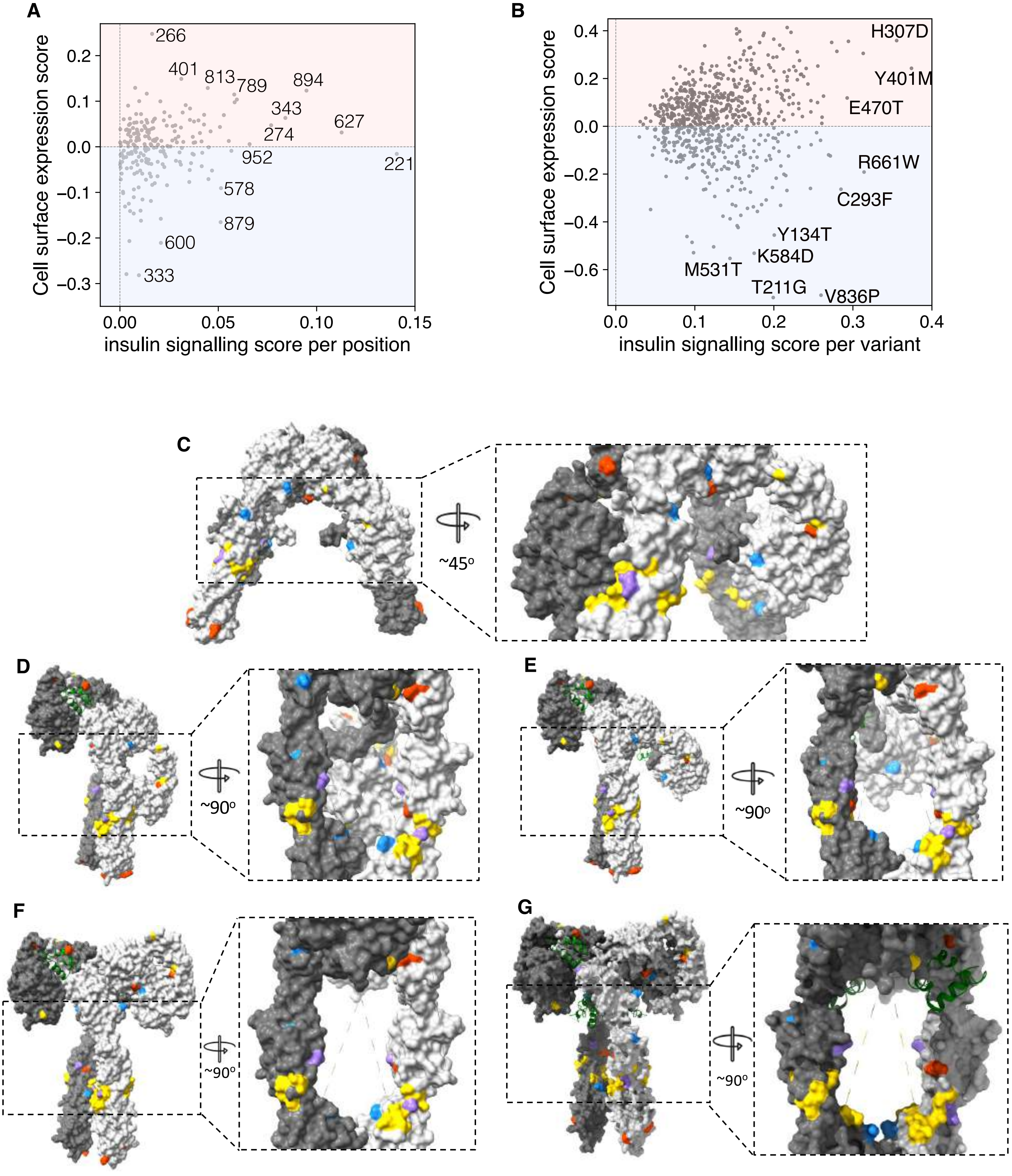

Supplementary Figure 9: Residues whose mutation confers gain of insulin signalling

See over for full figure legend

Supplementary Figure 9: Residues whose mutation confers gain of insulin signalling: A, Scatter plot of median expression scores per residue against insulin signalling scores, focused only on gain of signalling (score > 0). **B**, Scatter plot of significant gain-of-signalling scores (score > 0 and FDR <0.01) against expression scores for individual variants, including only variants with at least 3 replicate scores. **C–G,** INSR residues identified to result in gain of insulin binding and/or maximal insulin signalling upon substitution mapped to available cryo-EM structures of the various insulin-bound states. The two monomers are coloured different shades of grey. Insulin is represented by green ribbon structures. As in **Supplementary Figure 8**, yellow depicts residues where multiple substitutions result in gain of insulin binding (G183, H274, G333, D627, V631, N633, S634, S635, S636, W659, E660, R661, Q662, A663, E664, D665, S666, E667, S682, R683, T684, S686, P687, P688) and blue depicts residues where a unique substitution results in gain of insulin binding (D169, N541, N568, T653, K679). Added to these, orange depicts residues where a unique substitution confers gain of insulin-stimulated signalling (F258, C293, E343, N376, P576, Y839, F862, Y941). Purple depicts residues where substitution increases both insulin binding and signalling (N568C, D627, R661). Some of the flexible ID domain is not observed in the structures, and residues highlighted may be involved in interactions with parts of the receptor not resolved in available structures. **C,** Unbound, human INSR B-isoform (PDB: 8U4B). The highlighted section is rotated counterclockwise 45° to the full view. **D,** Single insulin bound (green ribbon) mouse INSR isoform A (PDB: 7STI). The highlighted section is rotated counterclockwise 90°. **E** and **F,** Two insulin bound (green ribbons) mouse INSR isoform A (PDB: 7STJ (asymmetrical conformation), and 7STH (symmetrical). The highlighted sections are rotated counterclockwise 90°. **G,** Saturated, four insulin bound (green ribbons) human INSR isoform A (PBD: 6PXV). The highlighted section is rotated counterclockwise 90°

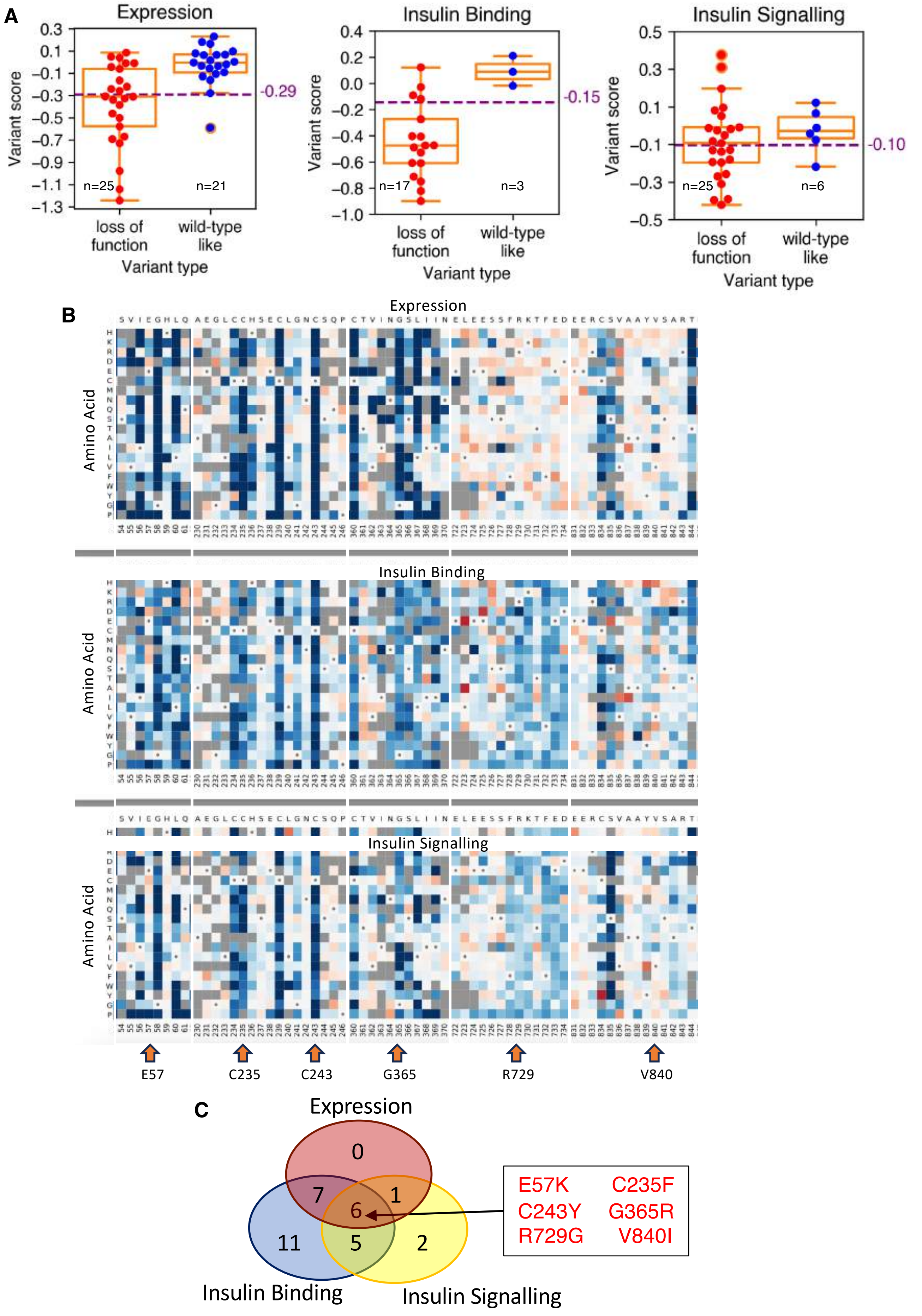

Supplementary Figure 10: Functionally impaired variants in ClinVar and/or gnomAD datasets: **A**, Boxplots of cell surface expression, insulin binding and insulin signalling scores for variants previously functionally studied (**Supplementary Data 3**). Boxes = interquartile range (IQR), with median line. Whiskers denote 1.5×IQR. The dashed horizontal line indicates the optimal cut off for binary classification of variants into wild-type and loss-of-function categories, determined through confusion matrix analysis. **B**, Variant Effect Map excerpts of 6 variants classified uniquely as VUS in ClinVar showing loss of function in all three assays. **C,** Venn diagram comparing results for categorical loss of expression, insulin binding, and signalling determined by applying cut-offs from A for variants classified uniquely as VUS in ClinVar.

Supplementary References

(Containing all Papers Cited in Supplementary Data 3 and 4)

1. Park, J. et al. Activation of the insulin receptor by an insulin mimetic peptide. *Nat Commun* **13**, 5594 (2022).
2. Uchikawa, E., Choi, E., Shang, G., Yu, H. & Bai, X.-C. Activation mechanism of the insulin receptor revealed by cryo-EM structure of the fully liganded receptor-ligand complex. *Elife* **8**, e48630 (2019).
3. Quon, M. J., Cama, A. & Taylor, S. I. Postbinding characterization of five naturally occurring mutations in the human insulin receptor gene: impaired insulin-stimulated c-jun expression and thymidine incorporation despite normal receptor autophosphorylation. *Biochemistry* **31**, 9947–9954 (1992).
4. van der Vorm, E. R. et al. An Arg for Gly substitution at position 31 in the insulin receptor, linked to insulin resistance, inhibits receptor processing and transport. *J Biol Chem* **267**, 66–71 (1992).
5. Jiang, S. et al. Functional characterization of insulin receptor gene mutations contributing to Rabson-Mendenhall syndrome - phenotypic heterogeneity of insulin receptor gene mutations. *Endocr J* **58**, 931–940 (2011).
6. Longo, N. et al. Genotype-phenotype correlation in inherited severe insulin resistance. *Hum Mol Genet* **11**, 1465–1475 (2002).
7. Longo, N., Langley, S. D., Griffin, L. D. & Elsas, L. J. Activation of glucose transport by a natural mutation in the human insulin receptor. *Proc Natl Acad Sci U S A* **90**, 60–64 (1993).
8. Maassen, J. A. et al. Identification and functional assessment of novel and known insulin receptor mutations in five patients with syndromes of severe insulin resistance. *J Clin Endocrinol Metab* **88**, 4251–4257 (2003).
9. Alzahrani, A. S. et al. Molecular characterization of a novel p.R118C mutation in the insulin receptor gene from patients with severe insulin resistance. *Clin Endocrinol (Oxf)* **76**, 540–547 (2012).
10. Brierley, G. V., Siddle, K. & Semple, R. K. Evaluation of anti-insulin receptor antibodies as potential novel therapies for human insulin receptoropathy using cell culture models. *Diabetologia* **61**, 1662–1675 (2018).
11. Raffan, E. et al. Founder effect in the Horn of Africa for an insulin receptor mutation that may impair receptor recycling. *Diabetologia* **54**, 1057–1065 (2011).
12. Carrera, P. et al. Substitution of Leu for Pro-193 in the insulin receptor in a patient with a genetic form of severe insulin resistance. *Hum Mol Genet* **2**, 1437–1441 (1993).
13. Maggi, D., Barbetti, F. & Cordera, R. Role of proline 193 in the insulin receptor post-translational processing. *Diabetologia* **42**, 435–442 (1999).
14. Kadowaki, T. et al. Five mutant alleles of the insulin receptor gene in patients with genetic forms of insulin resistance. *J Clin Invest* **86**, 254–264 (1990).
15. Klinkhamer, M. P. et al. A leucine-to-proline mutation in the insulin receptor in a family with insulin resistance. *EMBO J* **8**, 2503–2507 (1989).
16. Falik Zaccai, T. C. et al. Two novel mutations identified in familial cases with Donohue syndrome. *Mol Genet Genomic Med* **2**, 64–72 (2014).
17. Whitehead, J. P. et al. Multiple molecular mechanisms of insulin receptor dysfunction in a patient with Donohue syndrome. *Diabetes* **47**, 1362–1364 (1998).
18. Desbois-Mouthon, C. et al. Major circadian variations of glucose homeostasis in a patient with Rabson-Mendenhall syndrome and primary insulin resistance due to a mutation (Cys284-->Tyr) in the insulin receptor alpha-subunit. *Pediatr Res* **42**, 72–77 (1997).
19. Tuthill, A. et al. Functional characterization of a novel insulin receptor mutation contributing to Rabson-Mendenhall syndrome. *Clin Endocrinol (Oxf)* **66**, 21–26 (2007).
20. van der Vorm, E. R. et al. A mutation in the insulin receptor that impairs proreceptor processing but not insulin binding. *J Biol Chem* **269**, 14297–14302 (1994).
21. Kadowaki, T. et al. Two mutant alleles of the insulin receptor gene in a patient with extreme insulin resistance. *Science* **240**, 787–790 (1988).

22. Cama, A. et al. Two mutant alleles of the insulin receptor gene in a family with a genetic form of insulin resistance: a 10 base pair deletion in exon 1 and a mutation substituting serine for asparagine-462. *Hum Genet* **95**, 174–182 (1995).
23. Hosoe, J. et al. Structural Basis and Genotype-Phenotype Correlations of INSR Mutations Causing Severe Insulin Resistance. *Diabetes* **66**, 2713–2723 (2017).
24. Hart, L. M. et al. An insulin receptor mutant (Asp707 --> Ala), involved in leprechaunism, is processed and transported to the cell surface but unable to bind insulin. *J Biol Chem* **271**, 18719–18724 (1996).
25. Yoshimasa, Y. et al. Insulin-resistant diabetes due to a point mutation that prevents insulin proreceptor processing. *Science* **240**, 784–787 (1988).
26. Kadowaki, H. et al. Four mutant alleles of the insulin receptor gene associated with genetic syndromes of extreme insulin resistance. *Biochem Biophys Res Commun* **237**, 516–520 (1997).
27. Grasso, V. et al. Six cases with severe insulin resistance (SIR) associated with mutations of insulin receptor: Is a Bartter-like syndrome a feature of congenital SIR? *Acta Diabetol* **50**, 951–957 (2013).
28. Serrano Ríos, M. et al. Pancreatic A and B cell hyperfunction in the Mendenhall syndrome. *Diabetologia* **25**, 8–12 (1983).
29. Barbetti, F. et al. Detection of mutations in insulin receptor gene by denaturing gradient gel electrophoresis. *Diabetes* **41**, 408–415 (1992).
30. Maassen, J. A. et al. Improper expression of insulin receptors on fibroblasts from a leprechaun patient. *Eur J Biochem* **172**, 725–729 (1988).
31. Rouard, M. et al. Identification of two novel insulin receptor mutations, Asp59Gly and Leu62Pro, in type A syndrome of extreme insulin resistance. *Biochem Biophys Res Commun* **234**, 764–768 (1997).
32. Simpkin, A. et al. Insulin Receptor and the Kidney: Nephrocalcinosis in Patients with Recessive INSR Mutations. *Nephron Physiol* **128**, 55–61 (2014).
33. Takeuchi, T. et al. Clinical characteristics of insulin resistance syndromes: A nationwide survey in Japan. *J Diabetes Investig* **11**, 603–616 (2020).
34. Rojek, A., Wikiera, B., Noczynska, A. & Niedziela, M. Syndrome of Congenital Insulin Resistance Caused by a Novel INSR Gene Mutation. *J Clin Res Pediatr Endocrinol* **15**, 312–317 (2023).
35. Longo, N. et al. Genotype-phenotype correlation in inherited severe insulin resistance. *Hum Mol Genet* **11**, 1465–1475 (2002).
36. Huggard, D., Stack, T., Satas, S. & Gorman, C. O. Donohue syndrome and use of continuous subcutaneous insulin pump therapy. *BMJ Case Rep* 2015, bcr2015210019 (2015).
37. Ben Abdelaziz, R. et al. A novel homozygous missense mutation in the insulin receptor gene results in an atypical presentation of Rabson-Mendenhall syndrome. *Eur J Med Genet* **59**, 16–19 (2016).
38. Al-Kandari, H., Al-Abdulrazzaq, D., Al-Jaser, F., Al-Mulla, F. & Davidsson, L. Rabson-Mendenhall Syndrome in a brother-sister pair in Kuwait: Diagnosis and 5 year follow up. *Prim Care Diabetes* **15**, 175–177 (2021).
39. Ardon, O., Procter, M., Tvrdik, T., Longo, N. & Mao, R. Sequencing analysis of insulin receptor defects and detection of two novel mutations in INSR gene. *Mol Genet Metab Rep* **1**, 71–84 (2014).
40. Kushchayeva, Y. S. et al. Thyroid Abnormalities in Patients With Extreme Insulin Resistance Syndromes. *J Clin Endocrinol Metab* **104**, 2216–2228 (2019).
41. al-Gazali, L. I., Khalil, M. & Devadas, K. A syndrome of insulin resistance resembling leprechaunism in five sibs of consanguineous parents. *J Med Genet* **30**, 470–475 (1993).
42. Riqué, S. et al. Identification of three novel mutations in the insulin receptor gene in type A insulin resistant patients. *Clin Genet* **57**, 67–69 (2000).
43. Thiel, C. T. et al. Two novel mutations in the insulin binding subunit of the insulin receptor gene without insulin binding impairment in a patient with Rabson-Mendenhall syndrome. *Mol Genet Metab* **94**, 356–362 (2008).

44. Perge, K. et al. Intrauterine Growth Restriction and Hypertrophic Cardiomyopathy as Prenatal Ultrasound Findings in a Case of Leprechaunism. *Mol Syndromol* **11**, 223–227 (2020).
45. Mariani, S. et al. Insulin resistance in a child with Acanthosis nigricans type A. *Acta Paediatr Scand* **71**, 667–670 (1982).
46. Dozio, N. et al. In vivo demonstration of insulin-receptor defect with 123I-labeled insulin and scintigraphic scanning in severe insulin resistance. *Diabetes Care* **15**, 651–656 (1992).
47. Schilling, E. E., Rechler, M. M., Grunfeld, C. & Rosenberg, A. M. Primary defect of insulin receptors in skin fibroblasts cultured from an infant with leprechaunism and insulin resistance. *Proc Natl Acad Sci U S A* **76**, 5877–5881 (1979).
48. Rosenberg, A. M., Haworth, J. C., Degroot, G. W., Trevenen, C. L. & Rechler, M. M. A case of leprechaunism with severe hyperinsulinemia. *Am J Dis Child* **134**, 170–175 (1980).
49. Kim, D. et al. Two novel insulin receptor gene mutations in a patient with Rabson-Mendenhall syndrome: the first Korean case confirmed by biochemical, and molecular evidence. *J Korean Med Sci* **27**, 565–568 (2012).
50. Maassen, J. A. et al. Fibroblasts from a leprechaun patient have defects in insulin binding and insulin receptor autophosphorylation. *Diabetologia* **31**, 612–617 (1988).
51. Hacıhamdioğlu, B., Baş, E. G. & Delil, K. Homozygous Mutation in the Insulin Receptor Gene Associated with Mild Type A Insulin Resistance Syndrome: A Case Report. *J Clin Res Pediatr Endocrinol* **13**, 100–103 (2020).
52. de Kerdanet, M. et al. Ten-year improvement of insulin resistance and growth with recombinant human insulin-like growth factor 1 in a patient with insulin receptor mutations resulting in leprechaunism. *Diabetes Metab* **41**, 331–337 (2015).
53. Hamer, I. et al. An arginine to cysteine(252) mutation in insulin receptors from a patient with severe insulin resistance inhibits receptor internalisation but preserves signalling events. *Diabetologia* **45**, 657–667 (2002).
54. Nakashima, N., Umeda, F., Yanase, T. & Nawata, H. Insulin resistance associated with substitution of histidine for arginine 252 in the alpha-subunit of the human insulin receptor: trial of insulin-like growth factor I injection therapy to enhance insulin sensitivity. *J Clin Endocrinol Metab* **80**, 3662–3667 (1995).
55. Nakashima, N. et al. Type A-insulin resistance with lipopexia on extremities: a case report. *Endocrinol Jpn* **39**, 347–353 (1992).
56. Galderisi, A. et al. SGLT2i Improves Glycemic Control in Patients With Congenital Severe Insulin Resistance. *Pediatrics* **150**, e2021055671 (2022).
57. Melikyan, M. A. et al. [Donohue syndrome and use of continuous subcutaneous IGF1 pump therapy]. *Probl Endokrinol (Mosk)* 68, 79–86 (2022).
58. Mohanan, S. et al. Rabson-Mendenhall syndrome with recurrent cerebral infarcts caused by a novel INSR mutation. *Int J Dermatol* **52**, 182–185 (2013).
59. Krook, A. et al. Molecular scanning of the insulin receptor gene in syndromes of insulin resistance. *Diabetes* **43**, 357–368 (1994).
60. Kostopoulou, E., Shah, P., Ahmad, N., Semple, R. & Hussain, K. Gastrointestinal dysmotility and pancreatic insufficiency in 2 siblings with Donohue syndrome. Pediatr *Diabetes* **18**, 839–843 (2017).
61. Accili, D. et al. A mutation in the insulin receptor gene that impairs transport of the receptor to the plasma membrane and causes insulin-resistant diabetes. *EMBO J* **8**, 2509–2517 (1989).
62. Barnes, N. D., Palumbo, P. J., Hayles, A. B. & Folgar, H. Insulin resistance, skin changes, and virilization: a recessively inherited syndrome possibly due to pineal gland dysfunction. *Diabetologia* **10**, 285–289 (1974).
63. Kobayashi, M. et al. Insulin resistance due to a defect distal to the insulin receptor: demonstration in a patient with leprechaunism. *Proc Natl Acad Sci U S A* **75**, 3469–3473 (1978).
64. Kahn, C. R. et al. The syndromes of insulin resistance and acanthosis nigricans. Insulin-receptor disorders in man. *N Engl J Med* **294**, 739–745 (1976).

65. Rüdiger, H. W., Dreyer, M., Kühnau, J. & Bartelheimer, H. Familial insulin-resistant diabetes secondary to an affinity defect of the insulin receptor. *Hum Genet* **64**, 407–411 (1983).
66. Bamborschke, D. et al. Ultra-rapid emergency genomic diagnosis of Donahue syndrome in a preterm infant within 17 hours. *Am J Med Genet A* **185**, 90–96 (2021).
67. Brown, R. J., Cochran, E. & Gorden, P. Metreleptin improves blood glucose in patients with insulin receptor mutations. *J Clin Endocrinol Metab* **98**, E1749-1756 (2013).
68. Jiang, L. et al. [Leprechaunism: an inherited insulin resistance syndrome caused by the defect of insulin receptor]. *Zhonghua Nei Ke Za Zhi* **45**, 730–733 (2006).
69. Nobile, S., Semple, R. K. & Carnielli, V. P. A novel mutation of the insulin receptor gene in a preterm infant with Donohue syndrome and heart failure. *J Pediatr Endocrinol Metab* **25**, 363–366 (2012).
70. Hovnik, T. et al. Severe progressive obstructive cardiomyopathy and renal tubular dysfunction in Donohue syndrome with decreased insulin receptor autophosphorylation due to a novel INSR mutation. *Eur J Pediatr* **172**, 1125–1129 (2013).
71. Baqir, Z. S. et al. A novel leprechaunism mutation, Cys807Arg, in an Arab infant: a rare cause of hypoglycaemia. *Paediatr Int Child Health* **32**, 183–185 (2012).
72. Qin, L. et al. Novel heterozygous mutations of the INSR gene in a familial case of Donohue syndrome. *Clin Chim Acta* **473**, 26–31 (2017).
73. Moreira, R. O., Zagury, R. L., Nascimento, T. S. & Zagury, L. Multidrug therapy in a patient with Rabson-Mendenhall syndrome. *Diabetologia* **53**, 2454–2455 (2010).
74. Laimon, W. et al. Genetic and clinical heterogeneity of permanent neonatal diabetes mellitus: a single tertiary centre experience. *Acta Diabetol* **58**, 1689–1700 (2021).
75. Longo, N., Griffin, L. D., Shuster, R. C., Langley, S. & Elsas, L. J. Increased glucose transport by human fibroblasts with a heritable defect in insulin binding. *Metabolism* **38**, 690–697 (1989).
76. Abe, Y. et al. A case of Rabson-Mendenhall syndrome with a novel mutation in the tyrosine kinase domain of the insulin receptor gene complicated by medullary sponge kidney. *J Pediatr Endocrinol Metab* **25**, 587–590 (2012).
77. Kawashima, Y. et al. Leprechaunism (Donohue syndrome): a case bearing novel compound heterozygous mutations in the insulin receptor gene. *Endocr J* **60**, 107–112 (2013).
78. Pettersen E.F. et al UCSF ChimeraX: Structure visualization for researchers, educators, and developers. *Protein Sci.* **30**, 70-82 (2021).
79. Sparrow L.G. et al N-linked glycans of the human insulin receptor and their distribution over the crystal structure Proteins. **71**, 426-39 (2008).
80. Sparrow L.G. et al The location and characterisation of the O-linked glycans of the human insulin receptor Proteins **66**, 261-5 (2007).
81. Soos M.A. et al Monoclonal antibodies reacting with multiple epitopes on the human insulin receptor Biochem J 235, 199-208 (1986).
