# Supplementary material for: Deep mutational scanning of the human insulin receptor ectodomain to inform precision therapy for insulin resistance
Source: Nat Commun. 2025 Oct 15;16:9143. doi: 10.1038/s41467-025-64178-4 (PMC12528385; doi:10.1038/s41467-025-64178-4)
Supplement: Supplementary file 2 — Description of Additional Supplementary Files [file 41467_2025_64178_MOESM2_ESM.pdf]

Description of Additional Supplementary Files

Supplementary Data 1 (csv file): Barcode Scores Generated by all Experimental Replicates

Supplementary Data 2 (csv file): Variant Scores Computed for all Experimental Replicates

Supplementary Data 3 (Microsoft Excel document)**:** Curation of Prior Functional Studies of INSR Ectodomain Missense Variants

Supplementary Data 4 (Microsoft Excel document)**:** Curation of Published and New Cases with Pathogenic INSR Ectodomain Missense Variants

Supplementary Data 5 (Microsoft Excel document)**:** Missense Variants with Properties Amenable to Therapeutic Targeting by Monoclonal Antibodies 83-7 and 83-14

Supplementary Data 6 (Microsoft Excel document)**:** Primers and Oligonucleotides Used
