# Supplementary material for: Deep mutational scanning of the human insulin receptor ectodomain to inform precision therapy for insulin resistance
Source: Nat Commun. 2025 Oct 15;16:9143. doi: 10.1038/s41467-025-64178-4 (PMC12528385; doi:10.1038/s41467-025-64178-4)
Supplement: Supplementary file 9 — Reporting Summary [file 41467_2025_64178_MOESM9_ESM.pdf]

nature portfolio

Corresponding author(s): Robert SEMPLE

Last updated by author(s): Jul 15, 2025

Reporting Summary

Nature Portfolio wishes to improve the reproducibility of the work that we publish. This form provides structure for consistency and transparency in reporting. For further information on Nature Portfolio policies, see our Editorial Policies and the Editorial Policy Checklist.

Statistics

For all statistical analyses, confirm that the following items are present in the figure legend, table legend, main text, or Methods section.

n/a	Confirmed	
☐	☒	The exact sample size (n) for each experimental group/condition, given as a discrete number and unit of measurement
☒	☐	A statement on whether measurements were taken from distinct samples or whether the same sample was measured repeatedly
☐	☒	The statistical test(s) used AND whether they are one- or two-sided<br>*Only common tests should be described solely by name; describe more complex techniques in the Methods section.*
☒	☐	A description of all covariates tested
☐	☒	A description of any assumptions or corrections, such as tests of normality and adjustment for multiple comparisons
☐	☒	A full description of the statistical parameters including central tendency (e.g. means) or other basic estimates (e.g. regression coefficient) AND variation (e.g. standard deviation) or associated estimates of uncertainty (e.g. confidence intervals)
☐	☒	For null hypothesis testing, the test statistic (e.g. F, t, r) with confidence intervals, effect sizes, degrees of freedom and P value noted<br>*Give P values as exact values whenever suitable.*
☒	☐	For Bayesian analysis, information on the choice of priors and Markov chain Monte Carlo settings
☒	☐	For hierarchical and complex designs, identification of the appropriate level for tests and full reporting of outcomes
☐	☒	Estimates of effect sizes (e.g. Cohen's d, Pearson's r), indicating how they were calculated

Our web collection on statistics for biologists contains articles on many of the points above.

Software and code

Policy information about availability of computer code

Data collection	No software used for data collection
Data analysis	ChimeraX version 1.6-rc2023.04.05<br>alignparse version 0.6.3<br>minimap2 version 2.24<br>Enrich2<br>Custom Python Scrips available on https://doi.org/10.5281/zenodo.16788684

For manuscripts utilizing custom algorithms or software that are central to the research but not yet described in published literature, software must be made available to editors and reviewers. We strongly encourage code deposition in a community repository (e.g. GitHub). See the Nature Portfolio guidelines for submitting code & software for further information.

Data

Policy information about availability of data

All manuscripts must include a data availability statement. This statement should provide the following information, where applicable:

- Accession codes, unique identifiers, or web links for publicly available datasets
- A description of any restrictions on data availability
- For clinical datasets or third party data, please ensure that the statement adheres to our policy

The processed PacBio and illumina sequencing data are available at https://zenodo.org/records/16787091. Raw PacBio and illumina sequencing data generated in this study have been deposited in the GEO database under accession code GSE277112. Fully analysed data are also interrogatable in the curated MaveDB community database of MAVE data. Data can be found by searching for INSR from the MaveDB landing page (https://www.mavedb.org/). The Universal Resource Name (URN) for the dataset is urn:mavedb:00001239-a. Clicking on this URN leads to a project summary page that includes detailed metadata and links to individual score sets, each representing a distinct assay. Within each score set page, a heatmap is displayed showing variant scores spanning residues 28 to 955. In this heatmap, blue indicates variants with scores lower than wild type (WT), red indicates higher-than-WT scores, white represents scores approximately equal to WT (near zero), yellow marks the WT amino acid at each position, and black denotes positions for which no data are available. Above the heatmap, a histogram illustrates the distribution of scores for the corresponding assay. By default, MaveDB sets the midpoint of its heatmap colour scale to the median of the score distribution and interprets this value as the WT. Variants are then coloured on a red-white-blue gradient relative to this median. However, because our score distributions are asymmetric around zero (defined as WT), this default behaviour distorts the heatmap representation. To ensure accurate visualization, we rescaled the positive variant scores to match the scale of the negative scores, forcing the median to align with our WT at zero. This adjustment ensures scores below zero are correctly coloured blue, and scores above zero are consistently coloured red. To retrieve the score for a specific variant, users can enter the mutation using the three letter amino acid code (e.g., Cys35Lys) into the query box. This highlights the corresponding position in both the heatmap and distribution plot and displays its exact score. Each score set page also provides downloadable versions of the heatmap, histogram, and the full score file. The score files include not only the variant effect scores but also associated false discovery rate (FDR) values and replicate scores. The table below provides a summary of the score set URNs, their corresponding assays, and direct links (active as of July 2025).

MaveDB URN Assay Direct Link
urn:mavedb:00001239-a-7
Cell Surface Expression https://www.mavedb.org/score-sets/urn:mavedb:00001239-a-7
urn:mavedb:00001239-a-6
Insulin Binding https://www.mavedb.org/score-sets/urn:mavedb:00001239-a-6
urn:mavedb:00001239-a-1
Insulin Signalling https://www.mavedb.org/score-sets/urn:mavedb:00001239-a-1
urn:mavedb:00001239-a-2
mAb 83-07 Binding https://www.mavedb.org/score-sets/urn:mavedb:00001239-a-2
urn:mavedb:00001239-a-3
mAb 83-14 Binding https://www.mavedb.org/score-sets/urn:mavedb:00001239-a-3
urn:mavedb:00001239-a-4
mAb 83-14 Signalling https://www.mavedb.org/score-sets/urn:mavedb:00001239-a-4
urn:mavedb:00001239-a-5
mAb 83-07 Signalling https://www.mavedb.org/score-sets/urn:mavedb:00001239-a-5

Research involving human participants, their data, or biological material

Policy information about studies with human participants or human data. See also policy information about sex, gender (identity/presentation), and sexual orientation and race, ethnicity and racism.

Reporting on sex and gender	*Use the terms sex (biological attribute) and gender (shaped by social and cultural circumstances) carefully in order to avoid confusing both terms. Indicate if findings apply to only one sex or gender; describe whether sex and gender were considered in study design; whether sex and/or gender was determined based on self-reporting or assigned and methods used.*<br>*Provide in the source data disaggregated sex and gender data, where this information has been collected, and if consent has been obtained for sharing of individual-level data; provide overall numbers in this Reporting Summary. Please state if this information has not been collected.*<br>*Report sex- and gender-based analyses where performed, justify reasons for lack of sex- and gender-based analysis.*
Reporting on race, ethnicity, or other socially relevant groupings	*Please specify the socially constructed or socially relevant categorization variable(s) used in your manuscript and explain why they were used. Please note that such variables should not be used as proxies for other socially constructed/relevant variables (for example, race or ethnicity should not be used as a proxy for socioeconomic status).*<br>*Provide clear definitions of the relevant terms used, how they were provided (by the participants/respondents, the researchers, or third parties), and the method(s) used to classify people into the different categories (e.g. self-report, census or administrative data, social media data, etc.)*<br>*Please provide details about how you controlled for confounding variables in your analyses.*
Population characteristics	*Describe the covariate-relevant population characteristics of the human research participants (e.g. age, genotypic information, past and current diagnosis and treatment categories). If you filled out the behavioural & social sciences study design questions and have nothing to add here, write "See above."*

Recruitment	*Describe how participants were recruited. Outline any potential self-selection bias or other biases that may be present and how these are likely to impact results.*
Ethics oversight	*Identify the organization(s) that approved the study protocol.*

Note that full information on the approval of the study protocol must also be provided in the manuscript.

Field-specific reporting

Please select the one below that is the best fit for your research. If you are not sure, read the appropriate sections before making your selection.

☒ Life sciences ☐ Behavioural & social sciences ☐ Ecological, evolutionary & environmental sciences

For a reference copy of the document with all sections, see nature.com/documents/nr-reporting-summary-flat.pdf

Life sciences study design

All studies must disclose on these points even when the disclosure is negative.

Sample size	The total sample size included 17,632 possible mutations, of which we successfully obtained scores for at least 14,000 variants. The missing variants were primarily due to low mutagenesis coverage and insufficient barcode read counts from Illumina sequencing after FACS sorting.
Data exclusions	Barcodes with a total read count below 150 (summed across all four FACS bins) were excluded from the analysis. The mutagenesis was performed across amino acids 2 to 955 of the INSR protein. The puromycin selection marker is located downstream from the INSR gene, with a P2A self-cleaving sequence in between. Any stop codons in the INSR gene would stop translation of the Puromycin resistance gene and therefore cells carrying stop codons will be removed during antibiotic selection after transfection unless stop codon readthrough occurs. We expected no reads for such barcodes on Illumina sequencing but observed reads for 7% of the possible stop codon barcodes. We considered the possibility that these barcodes represent erroneous barcode-variant assignments in PacBio data analysis. To test this, we compared the counts of Illumina sequencing reads representing different classes of variants in FACS based assays. We found that barcodes associated with stop codons show greatly depleted read counts in the FACS data, compared to barcodes associated with wild-type, synonymous or missense variants. This strongly suggests that these barcodes represent bona fide stop codons that were only partially depleted during puromycin selection, perhaps thanks to stop codon readthrough, and supports the accuracy of our barcode-variant phasing approach.
Replication	two replicates for mAb83-7 binding, two replicates for mAb83-14 binding, two replicates for insulin binding, three replicates for mAb83-7 signalling, three replicates for mAb83-14 signalling, and five replicates for insulin signalling.
Randomization	*Describe how samples/organisms/participants were allocated into experimental groups. If allocation was not random, describe how covariates were controlled OR if this is not relevant to your study, explain why.*
Blinding	*Describe whether the investigators were blinded to group allocation during data collection and/or analysis. If blinding was not possible, describe why OR explain why blinding was not relevant to your study.*

Reporting for specific materials, systems and methods

We require information from authors about some types of materials, experimental systems and methods used in many studies. Here, indicate whether each material, system or method listed is relevant to your study. If you are not sure if a list item applies to your research, read the appropriate section before selecting a response.

Materials & experimental systems

n/a	Involved in the study	
☐	☒	Antibodies
☐	☒	Eukaryotic cell lines
☐	☐	Palaeontology and archaeology
☐	☐	Animals and other organisms
☐	☐	Clinical data
☐	☐	Dual use research of concern
☐	☐	Plants

Methods

n/a	Involved in the study	
☐	☐	ChIP-seq
☐	☒	Flow cytometry
☐	☐	MRI-based neuroimaging

Antibodies

Antibodies used	83-7 and 83-14 antibodies (gifts from Prof. Kenneth Siddle) were labelled with AF647 dye using Zip Alexa Fluor Rapid Antibody Labelling kit (Thermofisher, Z11235) following the manufacturer's protocol.<br><br>All the following antibodies are from Cell Signalling<br><br>AlexaFluor 647-conjugated anti-Phospho-Akt (Ser473/474) antibody, Catalogue number: 4075

Phospho-IGF-I Receptor β (Tyr1135/1136)/Insulin Receptor β (Tyr1150/1151) (19H7) Rabbit mAb, Catalogue number: 3024
Insulin Receptor β (4B8) Rabbit mAb, Catalogue number: 3025
Phospho-Akt (Ser473) (D9E) XP® Rabbit mAb , Catalogue number: 4060
Akt (pan) (40D4) Mouse mAb, Catalogue number: 2920
Phospho-p44/42 MAPK (Erk1) (Tyr204)/(Erk2) (Tyr187) (D1H6G) Mouse mAb, Catalogue number: 5726
p44/42 MAPK (Erk1/2) Antibody, Catalogue number: 9102
Anti-mouse IgG, HRP-linked Antibody, Catalogue number: 7076
Anti-rabbit IgG, HRP-linked Antibody, Catalogue number: 7074
Myc-Tag (9B11) Mouse mAb, Catalogue number: 2276
β-Actin Antibody, Catalogue number: 4967
IGF-I Receptor β (D23H3) XP® Rabbit mAb, Catalogue number: 9750

Validation

Antibodies validated as described in Supplementary Figure 1F.

Eukaryotic cell lines

Policy information about cell lines and Sex and Gender in Research

Cell line source(s)

Igf1r-/- mouse embryo fibroblasts (MEFs) were obtained from the Cosgrove laboratory CSIRO, Adelaide, Australia. Sell, C. et al. Effect of a null mutation of the insulin-like growth factor I receptor gene on growth and transformation of mouse embryo fibroblasts. Mol. Cell. Biol. 14, 3604–3612 (1994). Murine embryonic fibroblasts (MEFs) derived from the Igf1R-/- mouse line were infected at low multiplicity of infection with lentivirus encoding concatenated shRNAs targeting the Insr gene14, and exposed to 500ug/ml hygromycin-B for 2 weeks. Clonal cell lines were isolated by limiting serial dilution and screened for GFP expression upon doxycycline (DOX) addition. Four clones demonstrated strong inducible knockdown of endogenous Insr, one of which was used for all subsequent experiments (Supplementary Figure 3).
A previously described DOX-inducible BxB1 DNA recombinase landing pad (Tet-coBxb1-2A-BFP_IRES-iCasp9-2A-Blast_rtTA3, Addgene 171588) was introduced into R-MmINSR KD cells using the Lenti-X Packaging Single Shot (VSV-G) (Cat 631275) system. Briefly, 7µg of pLenti_Tet-coBxb1-2A-BFP_IRES-iCasp9-2A-Blast_rtTA3 lentiviral vector in 600ul water was mixed with Lenti-X Packaging plasmid and incubated at room temperature for 10 minutes. The mixture was transferred dropwise onto cultured Lenti-X 293T cells on a 10 cm plate and incubated at 37 °C in 5% CO_2. Medium was changed the next day and supernatant collected after 48 and 72 hrs and pooled. Collected medium was centrifuged (300x g, 5 min), and the supernatant filtered through a 0.45µm filter to remove debris. The landing pad construct expressed doxycycline-inducible blue fluorescent protein (BFP) and a Blasticidin resistance gene, which were used to confirm landing pad insertion. 100µl to 1ml lentiviral supernatant was used, and assessment of BFP expression 48 hours after infection was used to identify lines with an MOI <1. After treatment with 6 µg/ml Blasticidin for one week, cells with the highest BFP fluorescence were sorted into single cells in 96-well plates using a BD FACS Aria II (405-450/50 nm laser). Surviving clones were transferred into a 24-well plate and later 6-well plates. 400,000 of the 3 selected clonal lines were seeded on a 6 well plate and transfected with 2.5ug of attB-miRFP670 using Lipofectamine 3000. The medium was changed and 2ug/ml Puromycin added 48 hours after transfection. Cells were assessed for recombination after a week of antibiotic selection with a BD LSR Fortessa flow cytometer. One clone showing uniform loss of BFP and gain of miRFP670, indicative of a single landing pad was selected for use in subsequent experiments.

Authentication

To confirm efficient induction of expression of myc-tagged human INSR on exposure to doxycycline, and doxycycline-induced knockdown of endogenous mouse Insr, R- (Igf1r-/-), R-MmInsrKD, and R-MmInsrKD + HsINSRmyc cells were cultured in the presence or absence of 1ug/ml Doxycycline for 3 days at 37oC/5%CO_2. Cells were washed, snap frozen, and lysed on ice in lysis buffer (20mM HEPES, 150mM NaCl, 1.2mM $MgCl_2$, 1mM EGTA, 1mM PMST, 1mM Na_3VO_4, 10% v/v glycerol, 1% v/v Triton-X-100, Roche complete-EDTA protease inhibitors). Insoluble material was pelleted by centrifugation (10,000xg for 10min at 4oC) and supernatant quantified by BCA assay (ThermoFisher). 15µg lysate per lane was resolved on NuPAGE 4-12% Bis-Tris gels (ThermoFisher) and transferred to nitrocellulose by iBlotII (ThermoFisher). Membranes were blocked with 3% w/v BSA/TBST for 1hr at room temperature before overnight incubation at 4oC with primary antibodies from Cell Signalling Technologies (3025, 2276, 9750, 4967). Membranes were washed four times with 1xTBST prior to incubation with horseradish peroxidase (HRP)-conjugated secondary antibodies (Cell Signalling Technologies: 7076, 7074). Immobilon Western Chemiluminescent HRP substrate (Millipore) was used to detect protein-antibody complexes and grey-scale tag image file formats (TIFFs) captured utilising an iBright FL1500 Imaging System (ThermoFisher).

Mycoplasma contamination

Regularly tested every two weeks.

Commonly misidentified lines
(See ICLAC register)

Name any commonly misidentified cell lines used in the study and provide a rationale for their use.

Palaeontology and Archaeology

Specimen provenance

Provide provenance information for specimens and describe permits that were obtained for the work (including the name of the issuing authority, the date of issue, and any identifying information). Permits should encompass collection and, where applicable, export.

Specimen deposition	*Indicate where the specimens have been deposited to permit free access by other researchers.*
Dating methods	*If new dates are provided, describe how they were obtained (e.g. collection, storage, sample pretreatment and measurement), where they were obtained (i.e. lab name), the calibration program and the protocol for quality assurance OR state that no new dates are provided.*

☐ Tick this box to confirm that the raw and calibrated dates are available in the paper or in Supplementary Information.

Ethics oversight	*Identify the organization(s) that approved or provided guidance on the study protocol, OR state that no ethical approval or guidance was required and explain why not.*

Note that full information on the approval of the study protocol must also be provided in the manuscript.

Animals and other research organisms

Policy information about studies involving animals; ARRIVE guidelines recommended for reporting animal research, and Sex and Gender in Research

Laboratory animals	*For laboratory animals, report species, strain and age OR state that the study did not involve laboratory animals.*
Wild animals	*Provide details on animals observed in or captured in the field; report species and age where possible. Describe how animals were caught and transported and what happened to captive animals after the study (if killed, explain why and describe method; if released, say where and when) OR state that the study did not involve wild animals.*
Reporting on sex	*Indicate if findings apply to only one sex; describe whether sex was considered in study design, methods used for assigning sex. Provide data disaggregated for sex where this information has been collected in the source data as appropriate; provide overall numbers in this Reporting Summary. Please state if this information has not been collected. Report sex-based analyses where performed, justify reasons for lack of sex-based analysis.*
Field-collected samples	*For laboratory work with field-collected samples, describe all relevant parameters such as housing, maintenance, temperature, photoperiod and end-of-experiment protocol OR state that the study did not involve samples collected from the field.*
Ethics oversight	*Identify the organization(s) that approved or provided guidance on the study protocol, OR state that no ethical approval or guidance was required and explain why not.*

Note that full information on the approval of the study protocol must also be provided in the manuscript.

Clinical data

Policy information about clinical studies

All manuscripts should comply with the ICMJEguidelines for publication of clinical research and a completedCONSORT checklist must be included with all submissions.

Clinical trial registration	*Provide the trial registration number from ClinicalTrials.gov or an equivalent agency.*
Study protocol	*Note where the full trial protocol can be accessed OR if not available, explain why.*
Data collection	*Describe the settings and locales of data collection, noting the time periods of recruitment and data collection.*
Outcomes	*Describe how you pre-defined primary and secondary outcome measures and how you assessed these measures.*

Dual use research of concern

Policy information about dual use research of concern

Hazards

Could the accidental, deliberate or reckless misuse of agents or technologies generated in the work, or the application of information presented in the manuscript, pose a threat to:

No	Yes	
☒	☐	Public health
☒	☐	National security
☒	☐	Crops and/or livestock
☒	☐	Ecosystems
☒	☐	Any other significant area

Experiments of concern

Does the work involve any of these experiments of concern:

No	Yes	
☒	☐	Demonstrate how to render a vaccine ineffective
☒	☐	Confer resistance to therapeutically useful antibiotics or antiviral agents
☒	☐	Enhance the virulence of a pathogen or render a nonpathogen virulent
☒	☐	Increase transmissibility of a pathogen
☒	☐	Alter the host range of a pathogen
☒	☐	Enable evasion of diagnostic/detection modalities
☒	☐	Enable the weaponization of a biological agent or toxin
☒	☐	Any other potentially harmful combination of experiments and agents

Plants

Seed stocks

Report on the source of all seed stocks or other plant material used. If applicable, state the seed stock centre and catalogue number. If plant specimens were collected from the field, describe the collection location, date and sampling procedures.

Novel plant genotypes

Describe the methods by which all novel plant genotypes were produced. This includes those generated by transgenic approaches, gene editing, chemical/radiation-based mutagenesis and hybridization. For transgenic lines, describe the transformation method, the number of independent lines analyzed and the generation upon which experiments were performed. For gene-edited lines, describe the editor used, the endogenous sequence targeted for editing, the targeting guide RNA sequence (if applicable) and how the editor was applied.

Authentication

Describe any authentication procedures for each seed stock used or novel genotype generated. Describe any experiments used to assess the effect of a mutation and, where applicable, how potential secondary effects (e.g. second site T-DNA insertions, mosiacism, off-target gene editing) were examined.

ChIP-seq

Data deposition

☐ Confirm that both raw and final processed data have been deposited in a public database such as GEO.

☐ Confirm that you have deposited or provided access to graph files (e.g. BED files) for the called peaks.

Data access links
May remain private before publication.

For "Initial submission" or "Revised version" documents, provide reviewer access links. For your "Final submission" document, provide a link to the deposited data.

Files in database submission

Provide a list of all files available in the database submission.

Genome browser session
(e.g. UCSC)

Provide a link to an anonymized genome browser session for "Initial submission" and "Revised version" documents only, to enable peer review. Write "no longer applicable" for "Final submission" documents.

Methodology

Replicates

Describe the experimental replicates, specifying number, type and replicate agreement.

Sequencing depth

Describe the sequencing depth for each experiment, providing the total number of reads, uniquely mapped reads, length of reads and whether they were paired- or single-end.

Antibodies

Describe the antibodies used for the ChIP-seq experiments; as applicable, provide supplier name, catalog number, clone name, and lot number.

Peak calling parameters

Specify the command line program and parameters used for read mapping and peak calling, including the ChIP, control and index files used.

Data quality

Describe the methods used to ensure data quality in full detail, including how many peaks are at FDR 5% and above 5-fold enrichment.

Software

Describe the software used to collect and analyze the ChIP-seq data. For custom code that has been deposited into a community repository, provide accession details.

Flow Cytometry

Plots

Confirm that:

- [x] The axis labels state the marker and fluorochrome used (e.g. CD4-FITC).
- [x] The axis scales are clearly visible. Include numbers along axes only for bottom left plot of group (a 'group' is an analysis of identical markers).
- [x] All plots are contour plots with outliers or pseudocolor plots.
- [x] A numerical value for number of cells or percentage (with statistics) is provided.

Methodology

Sample preparation	Cells harvested, and labelled by antibodies and washed 4X times PBS and resuspended in PBS for FACS sorting
Instrument	BD FACSAria™ II Cell Sorter
Software	BD FACSDiva™ Software V9
Cell population abundance	A BD FACS Aria II sorter was gated for intact cells and singlets, and cells were sorted into four bins by fluorescence in the 640-670/14A channel. At least 13 million cells were collected per bin. Sorted cells were collected by centrifugation and stored at -20°C before gDNA extraction and library preparation.
Gating strategy	To validate FACS-based assays of INSR expression and function, we used non transfected cells, and cells transfected with either wild-type (WT) INSR or the mutant INSR library. Both monoclonal antibodies used robustly detected surface INSR expression in WT and library cells compared to untransfected cells (Figures 1C, S1F). Similarly, binding of labelled insulin produced comparable profiles for WT and library cells which were clearly distinct from the profile of untransfected cells, although some insulin binding was detected in control cells, consistent with low level residual expression of murine Insr.<br>A smaller dynamic range was observed in assays for phosphorylated AKT (pAKT) (Figures 1C, S1F), as expected for a labile intracellular antigen. Shifts in pAKT FACS profile were seen on stimulation by insulin, 83-7 or 83-14 antibodies for both WT and library cells, but some pAKT was also detected on insulin stimulation of untransfected cells. The lack of pAkt detection in untransfected cells stimulated with human INSR-specific antibodies further supports this being due to binding to endogenous mouse Insr, in keeping with the residual insulin binding noted above. We also observed that overexpression of either WT or mutated human INSR induced some AKT phosphorylation even without stimulation. However, despite these limitations of the cellular system used, we consistently observed much stronger binding of and signalling by insulin and antibodies than background.

- [x] Tick this box to confirm that a figure exemplifying the gating strategy is provided in the Supplementary Information.

Magnetic resonance imaging

Experimental design

Design type	*Indicate task or resting state; event-related or block design.*
Design specifications	*Specify the number of blocks, trials or experimental units per session and/or subject, and specify the length of each trial or block (if trials are blocked) and interval between trials.*
Behavioral performance measures	*State number and/or type of variables recorded (e.g. correct button press, response time) and what statistics were used to establish that the subjects were performing the task as expected (e.g. mean, range, and/or standard deviation across subjects).*

Acquisition

Imaging type(s)	*Specify: functional, structural, diffusion, perfusion.*
Field strength	*Specify in Tesla*
Sequence & imaging parameters	*Specify the pulse sequence type (gradient echo, spin echo, etc.), imaging type (EPI, spiral, etc.), field of view, matrix size, slice thickness, orientation and TE/TR/flip angle.*
Area of acquisition	*State whether a whole brain scan was used OR define the area of acquisition, describing how the region was determined.*

Diffusion MRI ☐ Used ☐ Not used

Preprocessing

Preprocessing software	*Provide detail on software version and revision number and on specific parameters (model/functions, brain extraction, segmentation, smoothing kernel size, etc.).*
Normalization	*If data were normalized/standardized, describe the approach(es): specify linear or non-linear and define image types used for transformation OR indicate that data were not normalized and explain rationale for lack of normalization.*
Normalization template	*Describe the template used for normalization/transformation, specifying subject space or group standardized space (e.g. original Talairach, MNI305, ICBM152) OR indicate that the data were not normalized.*
Noise and artifact removal	*Describe your procedure(s) for artifact and structured noise removal, specifying motion parameters, tissue signals and physiological signals (heart rate, respiration).*
Volume censoring	*Define your software and/or method and criteria for volume censoring, and state the extent of such censoring.*

Statistical modeling & inference

Model type and settings	*Specify type (mass univariate, multivariate, RSA, predictive, etc.) and describe essential details of the model at the first and second levels (e.g. fixed, random or mixed effects; drift or auto-correlation).*
Effect(s) tested	*Define precise effect in terms of the task or stimulus conditions instead of psychological concepts and indicate whether ANOVA or factorial designs were used.*

Specify type of analysis: ☐ Whole brain ☐ ROI-based ☐ Both

Statistic type for inference<br>(See Eklund et al. 2016)	*Specify voxel-wise or cluster-wise and report all relevant parameters for cluster-wise methods.*
Correction	*Describe the type of correction and how it is obtained for multiple comparisons (e.g. FWE, FDR, permutation or Monte Carlo).*

Models & analysis

n/a	Involved in the study
☐	☐ Functional and/or effective connectivity
☐	☐ Graph analysis
☐	☐ Multivariate modeling or predictive analysis

Functional and/or effective connectivity	*Report the measures of dependence used and the model details (e.g. Pearson correlation, partial correlation, mutual information).*
Graph analysis	*Report the dependent variable and connectivity measure, specifying weighted graph or binarized graph, subject- or group-level, and the global and/or node summaries used (e.g. clustering coefficient, efficiency, etc.).*
Multivariate modeling and predictive analysis	*Specify independent variables, features extraction and dimension reduction, model, training and evaluation metrics.*
